# Rift Valley Fever and a New Paradigm of Research and Development for Zoonotic Disease Control 

**DOI:** 10.3201/eid1902.120941

**Published:** 2013-02

**Authors:** Osman Dar, Sabrina McIntyre, Sue Hogarth, David Heymann

**Affiliations:** Author affiliations: London School of Hygiene and Tropical Medicine, London, UK (O. Dar, S. McIntyre, S. Hogarth);; Chatham House Centre on Global Health Security, London (D. Heymann); London School of Hygiene and Tropical Medicine, London (D. Heymann)

**Keywords:** Rift Valley fever, communicable disease control global, research and development, social justice, equity, viruses, zoonoses

## Abstract

Although Rift Valley fever is a disease that, through its wider societal effects, disproportionately affects vulnerable communities with poor resilience to economic and environmental challenge, Rift Valley fever virus has since its discovery in 1931 been neglected by major global donors and disease control programs. We describe recent outbreaks affecting humans and animals and discuss the serious socioeconomic effects on the communities affected and the slow pace of development of new vaccines. We also discuss the mixed global response, which has largely been fueled by the classification of the virus as a potential bioterrorism agent and its potential to migrate beyond its traditional eastern African boundaries. We argue for a refocus of strategy with increased global collaboration and a greater sense of urgency and investment that focuses on an equity-based approach in which funding and research are prioritized by need, inspired by principles of equity and social justice.

## Abstract 2

To control this disease, funding and research should be prioritized on the basis of determined needs.

Since Rift Valley fever virus (RVFV) was first identified in 1931, after an investigation of an epizootic among sheep on a farm in the Great Rift Valley of Kenya, the understanding of this zoonotic disease has grown considerably (*1*). With the rapid progress of molecular biology and genetic techniques in recent years, studies of prevailing circulating variants of RVFV have pointed to a recent common ancestor that existed during 1880–1890. This finding lends weight to the predominant hypothesis on the origins of human outbreaks of Rift Valley fever, which suggests that the development of industrialized agriculture systems and the introduction of highly susceptible European breeds of livestock into East Africa during the colonial era led to amplification of the virus in animal and arthropod vectors and may have been responsible for the establishment of the disease (*2*).

Although the disease disproportionately affects vulnerable communities with low resilience to economic and environmental challenges, RVF has remained largely neglected by major global donors and disease control programs. With high numbers of competent vector species present in disease-free regions, the intensification of international trade in live animals, and the uncertain effects of climate change, RVF is now considered a major challenge in global zoonotic disease control (*2*).

## Recent Outbreaks

The potential of RVFV to migrate was established after large outbreaks of RVF occurred among animals and humans in Egypt in 1977, in other geographic zones of Africa, and then outside the African continent in Saudi Arabia and Yemen in 2000 (*3*,*4*). The Figure illustrates how the disease has traveled away from its original identified location in humans and animals.

**Figure:**
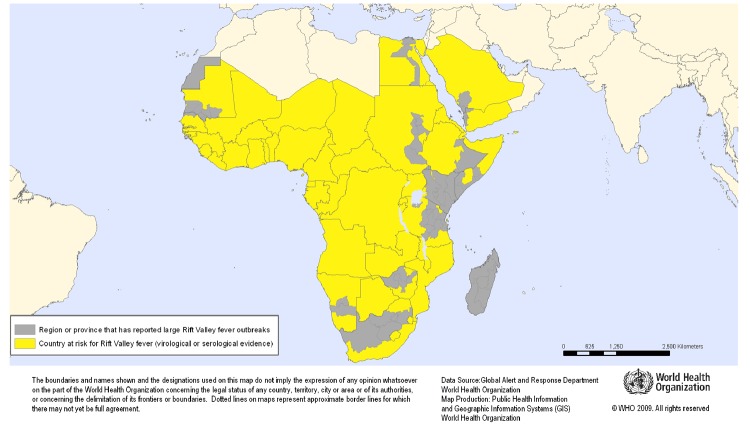
Geographic distribution of Rift Valley fever outbreaks in animals and humans, 1997–2010 (*5*).

The Table further demonstrates the spread of the disease; 7 of 9 major outbreaks in the past 15 years resulted in human cases outside the Rift Valley region in East Africa. The Table also highlights the difficulty of developing adequate surveillance systems and therefore the difficulty of accurately estimating morbidity and mortality rates for human populations in resource-poor settings. In the 5 outbreaks for which estimated numbers of human cases have been published, ≈339,000 infections are believed to have occurred. In the 4 outbreaks for which estimated and reported cases are documented, numbers of estimated cases are 78× higher than numbers of reported cases (Table). This difference between estimated and known numbers of cases highlights the inherent complexity of managing outbreaks, monitoring their spread, and mitigating their effects.

**Table:** Major Rift Valley fever outbreaks and reported cases among humans, 1997–2010*

Outbreak dates	Geographic distribution	Estimated no. cases	No. cases reported	No. deaths confirmed	Precipitation	Control measures
1997 Dec–1998 Jan	Kenya, Somalia, Tanzania	89,000	No documented reports	478	Heavy rainfall and flooding	Active surveillance; safety education; distribution of masks, gloves; slaughterhouse monitoring
1998 Sep–Dec	Mauritania	No documented estimates	300–400	6	Heavy rainfall	Active surveillance; public awareness/education; mosquito control; animal movement control
2000 Aug–2001 Sep	Saudi Arabia, Yemen	20,000†	886	123	Rainfall; virus introduction	Active surveillance; public awareness/education; mosquito control; animal movement control
2006 Nov–2007 Mar	Kenya	75,000	700	158	Heavy rainfall and flooding	Active surveillance; public awareness/education; mosquito control; ban on livestock slaughtering; closure of livestock market; vaccination Jan 2007
	Somalia	30,000	114	51		
	Tanzania	40,000	264	109		
2007 Sep–2008 Jan	Sudan	75,000	747	230	Heavy rainfall and flooding	Active surveillance; public awareness/education; targeted vaccination; ban of livestock imports by Saudi Arabia and Egypt
2008 Jan–Jun	Madagascar	10,000	476	19	Heavy rainfall	Active surveillance; public awareness; mosquito control; animal movement control
2008 Oct–2009 May	Madagascar	No documented estimates	236	7	Heavy rainfall	Active surveillance; public awareness; mosquito control; animal movement control
2010 Feb–2010 May	South Africa	No documented estimates	242	26	Sustained heavy rains	Public awareness/education; mosquito control
2010 Sep–2010 Dec	Mauritania	No documented estimates	63	13	Heavy rainfall	Public awareness; mosquito control; animal movement control

*Sources: (*6**–**24*). FAO, Food and Agriculture Organization of the United Nations. †Data available for Jizan region only.

## Socioeconomic Effects

There is a paucity of studies that have examined the socioeconomic effects of past outbreaks of RVFV, which reflects a lack of research focus on the broader social effects of the disease. One study that did examine the socioeconomic effects of the 2006/2007 RVFV outbreak in Kenya highlighted the concern that the outbreak had tended to disproportionately affect impoverished pastoralist communities, with those in the North Eastern Province of Kenya being hardest hit (*25*,*26*). The lack of understanding of the epidemiology and pathophysiology of RVFV, poor compliance with international health and safety standards by animal exporters, and the limited options for prevention and treatment have periodically led to summary bans of imports of livestock from disease-endemic areas.

The ban of livestock imports to the Middle East from East Africa, instituted after the 1997/1998 RVFV outbreak in Kenya and Somalia, particularly affected the export trade out of Somalia. The ban was variably enforced by several Middle Eastern countries but most notably by Saudi Arabia, which imports large numbers of ruminants for the annual Hajj pilgrimage. In 1997, the year before the onset of the ban, 2.8 million live animals were exported from the Somaliland port of Berbera, making it the single biggest exporting port for ruminants in the world that year. With the livestock trade accounting for 65% of gross domestic product in Somaliland, the export ban had a devastating effect on a region already suffering in the grip of a protracted civil war (*27*). Estimated losses from export sales out of Somaliland alone for the first 16 months of the ban from February 1998 to May 1999 totaled $109 million (*28*). By the time the ban on animal imports was lifted by Saudi Arabia in 2009, this drought- and war-affected region of East Africa had already endured many years of lost income because of prevailing fears concerning RVFV.

## Vaccine Development and Production

The slow pace of development of new vaccines (Technical Appendix) and diagnostic kits for RVFV and the limited supplies and relatively high cost of those currently available mean that there is a chronic worldwide shortage and lack of availability in areas where they are most needed. To address these problems, a “pull” strategy has been suggested by the Food and Agriculture Organization of the United Nations for the development of new RVFV vaccines. In this model, governments commit themselves to buy, at an agreed-upon price, whichever vaccine meets predefined requirements, thus giving an incentive to pharmaceutical companies to pursue the development of the most promising vaccine candidate. However, this strategy does not seem to be having much effect in the short term; the current financial crisis is limiting the purchasing power of national governments (*29*).

Before modern safety standards were instituted in laboratories, RVFV was regularly transmitted between laboratory staff; 47 cases were documented worldwide (*30*,*31*). International regulations for working with the live virus, and particularly for the production of vaccine and diagnostic test kits, typically require biosafety level 3 (BSL-3) laboratory facilities as a minimum and enhanced BSL-3 Ag/ABSL-3 (with many of the features of a BSL-4 laboratory) for working with live RVFV and loose-housed animals (*30*).

Fortunately, with the advent of recombinant genetic technology and the development of reverse transcription PCR techniques obviating the need to handle and store live virus, new vaccines and diagnostic tests in development can now be produced in laboratories of lower BSL (*29*,*32*). However, for the standard techniques that do involve storage and handling of live virus, because no reported laboratory infections have occurred since modern standard infection control procedures were introduced in the early 1980s (*33*), the case could be made for lowering currently prescribed BSL requirements. If laboratory workers handling live virus in these settings are all vaccinated, the laboratories required could possibly be reduced to BSL-2 with controlled access in disease-endemic countries, and to BSL-2 with controlled access and additional enhancements for working with animals in non–disease-endemic countries. Such a change could lower global production costs of vaccines and diagnostic tests and increase their accessibility by communities most affected by RVFV.

## Global Interest, Challenges, and Cooperation

Interest in RVFV and investment in its control were only substantially increased among the global health research and policy community after greater awareness of its potential to migrate beyond its traditional East African boundaries was noted. However, the recognition that much of the industrialized world has animals and arthropod vectors capable of transmitting the virus seems to have focused and accelerated efforts to develop improved tools for outbreak forecasting, monitoring, diagnosis, and prevention.

In more recent years, the classification of the virus as a potential bioterrorism/agroterrorism agent has also helped spur investment and activity, particularly in the area of vaccine development and diagnostics (*34*). Although this theoretical risk has contributed to increased funding over the past few decades, most notably from military sources such as the US Army Medical Research Institute of Infectious Diseases, this concern might also have acted as an impediment to the collaborative aspects of this high-quality work, with research being restricted to fewer, more expensive laboratories (*35*).

Growing restrictions stemming from biosecurity concerns now affect research activity across a range of infectious diseases and have most recently been highlighted by concerns over the publication of research into the production of genetically engineered variants of the influenza A subtype H5N1 virus (*36*). Limiting the dissemination of such research findings could, in any case, curtail technology transfer crucial to studying viruses such as RVFV and could theoretically cause expert technical knowledge and skills to be less accessible. This possibility not only has the potential to delay progress in developing new treatments and vaccines but could also increase their costs by limiting where they could be produced, resulting in decreased production capacity and competition.

Increased sales costs of vaccines have a variety of negative consequences; in particular, this increase could put at risk well-established mechanisms of international cooperation in global infectious disease surveillance. This risk was dramatically highlighted in 2006 and 2007 when Indonesia refused to share samples of influenza subtype H5N1 isolates with the World Health Organization. The event caused a risk to global health and occurred in direct protest to the inequitable sharing of virus samples and vaccine development technology (*37*).

Despite some of these challenges, some positive developments have occurred in global collaborative efforts for controlling zoonotic diseases, including RVFV. These include initiatives like the One Health (*38*) approach of integrating animal and human health challenges and the closer integration of multilateral agencies such as the World Organisation for Animal Health, the Food and Agriculture Organization of the United Nations, and the World Health Organization. These efforts have already resulted in improved outbreak forecasting and surveillance of RVFV in humans and animals, facilitated by the development of initiatives such as the Global Early Warning System (*39*,*40*). In addition, the rapid increase in socioeconomic interest and investment in RVFV-affected regions of Africa from emerging economies such as the People’s Republic of China, and Middle Eastern countries such as Saudi Arabia, provides an opportunity for their increased involvement in, and funding of, RVFV control. Countries benefiting from this socioeconomic interest and investment should develop the necessary information base and negotiating skill to successfully ensure that funds are channeled to such opportunities.

## Conclusion

In recent years, the perceived risk of RVFV becoming established in Europe and North America, and the theoretical risk of it being used as a bioterrorism agent, has brought a welcomed increase in investment to combat the disease yet has skewed priority areas of focus for that investment. The ideal that should be adopted is a more equity-based approach in which funding and research are prioritized on a needs-identified basis for the aid of those most disadvantaged in the global community. This approach would concentrate efforts on those interventions that most positively affect these vulnerable communities and, in addition, prevent or minimize the spread of the disease to previously non–disease-endemic high-income countries.

Such an approach would ensure research and policy emphasis on the socioeconomic effects of RVFV outbreaks. Interventions could then address international trade policies and their ramifications on livestock trade and the development of appropriate support systems within exporting countries to mitigate and minimize the risk of bans being instituted. In addition, encouraging farmers to focus their livestock-rearing efforts on breeds more resistant to infection with RVFV and a greater study of the genetic factors that make these breeds resistant should also be promoted as part of this global effort. Developing better surveillance systems is key.

Fears of RVFV being used as a bioterrorism agent should not sideline the real security effects of the disease in driving impoverished communities to find other, more dangerous means of income. Did the bans on livestock from Somalia, for instance, and the resulting lost economic opportunities afforded by a well-developed functioning ruminant export market, contribute to the drive of persons and communities to seek alternative sources of income, including taking part as combatants in the civil war in or in the piracy trade that has developed in the region? Are the stringent measures being imposed on laboratories that store or work with the virus serving to concentrate technical expertise and industrial know-how in the hands of scientists in a very few industrialized countries, thus contributing to limited scientific inquiry and collaboration, which further escalates costs? Although these questions are yet to be answered conclusively, exploring the case for lowering current BSL requirements of laboratories and production facilities could be 1 method of mitigating these costs.

A greater sense of urgency and investment is required for controlling, better managing, and preventing future large-scale outbreaks of RVFV. Future long-term success lies in building on global collaborative initiatives, the closer integration of multilateral agencies, and a wider participation from livestock-importing countries and emerging economies that are investing in RVFV-endemic countries. A worldwide strategy, both in tune with and inspired by principles of equity and social justice, could ultimately deliver the best outcomes in combating this neglected tropical disease.

Technical Appendix Rift Valley fever vaccine development.
