# Supplementary material for: Rift Valley Fever and a New Paradigm of Research and Development for Zoonotic Disease Control
Source: Emerg Infect Dis. 2013 Feb;19(2):189–93. doi: 10.3201/eid1902.120941 (PMC3559053; doi:10.3201/eid1902.120941)
Supplement: Technical Appendix — Rift Valley fever vaccine development. [file 12-0941-Techapp-s1.pdf]

Article DOI: http://dx.doi.org/10.3201/eid1902.120941

# Rift Valley Fever and a New Paradigm of Research and Development for Zoonotic Disease Control

## Technical Appendix

### Vaccine Development

The first major breakthrough in efforts to combat Rift Valley fever (RVF) in animals occurred with the development of the live-attenuated Smithburn vaccine in the late 1940s. Although it was effective in inducing long-term immunity in animals, the vaccine unfortunately retains some virulence and is teratogenic, causing abortions in ewes, cows, and goats. This characteristic and the associated fears of inadvertently spreading RVF virus through needle transmission in animals during an outbreak has led to its use being restricted to nonpregnant animals preceding an imminent outbreak. A later inactivated-virus vaccine developed in the 1960s, although it did not have the same deleterious side-effect profile as the Smithburn strain, remains prohibitively expensive to prepare and requires 3 annually spaced inoculations to provide adequate immunity. Therefore, it is logistically complicated to deliver in low-income settings.

Several newer candidate vaccines have been in development since the 1970s, most notably the live attenuated strains MP-12 and clone 13 and, more recently, the R566 strain. The slow pace of their development, a process that has continued over 3 decades, highlights the relative lack of investment and the constrained nature of the research into this restricted pathogen. A positive development in this regard is the recent introduction of clone 13 for use in South Africa after studies demonstrated that the vaccine does not, seemingly, retain any of the residual pathogenicity or teratogenicity of the Smithburn vaccine.

Although an inactivated human vaccine strain, TSI-GSD-200, is available for use in limited quantities for military and laboratory staff, no vaccine is available for routine, generalized use in humans. Advances in technology and increased investment in the last decade

have meant that there is now a promising array of candidates for development, including recombinant vaccines, DNA based vaccines, and viruslike-particle vaccines. However, because relatively few laboratories and organizations have the facilities and skills to develop these vaccines, prioritizing their development over other more lucrative infections and vaccines remains problematic (*1*–*3*).

**References**

1. Daubney R, Hudson JR, Garnham PC. Enzootic hepatitis or Rift Valley fever. An undescribed virus disease of sheep, cattle and man from East Africa. J Pathol Bacteriol. 1931;34:545–79. http://dx.doi.org/10.1002/path.1700340418

2. Food and Agriculture Organization of the United Nations. Rift Valley fever vaccine development, progress and constraints. Proceedings of the Global Framework for the Progressive Control of Transboundary Animal Diseases. January 2011; Rome, Italy [cited 2012 Dec 3]. http://www.fao.org/docrep/014/i2310e/i2310e00.pdf

3. Boshra H, Lorenzo G, Busquets N, Brun A. Rift valley fever: recent insights into pathogenesis and prevention. J Virol. 2011;85:6098–105.
